# Air Pollution Exposure and Risk of Spontaneous Pneumothorax in Children: A Longitudinal, Nationwide Study

**DOI:** 10.3390/children9010061

**Published:** 2022-01-04

**Authors:** Jing-Cheng Wang, Cheng-Li Lin, Chieh-Ho Chen, Chien-Heng Lin

**Affiliations:** 1 Division of Pediatrics Pulmonology, China Medical University Children’s Hospital, Taichung 404327, Taiwan; fcwelldone@gmail.com (J.-C.W.); d30270@mail.cmuh.org.tw (C.-H.C.); 2 Management Office for Health Data, China Medical University Hospital, Taichung 404327, Taiwan; orangechengli@gmail.com; 3 Department of Biomedical Imaging and Radiological Science, College of Medicine, China Medical University, Taichung 404328, Taiwan

**Keywords:** air pollution, spontaneous pneumothorax, children, nitric oxides, hydrocarbons

## Abstract

Spontaneous pneumothorax (SP) involves the spontaneous appearance of air in the pleural space. Atmospheric pressure, temperature change, and seasonal factors may precipitate SP, but its association with air pollution remains unclear. Therefore, we conducted this nationwide, retrospective population-based study to evaluate the risk of SP in Taiwanese children exposed to air pollution. We collected data on SP incidence from the Longitudinal Health Insurance Database; the Taiwan Air Quality-Monitoring Database provided daily concentrations of nitric oxide (NO), nitrogen dioxide (NO_2_), and hydrocarbons in 2000–2012. SP risk was evaluated for four quartiles (Q1, Q2, Q3, Q4). The NO adjusted hazard ratios (aHRs) for Q2, Q3, and Q4 compared to Q1 were 1.11 (95% confidence interval (CI): 0.77–1.61), 1.24 (95% CI: 0.88–1.76), and 1.66 (95% CI: 1.17–2.34), respectively. The NO_2_ aHRs for Q2, Q3, and Q4 were 1.12 (95% CI: 0.77–1.64), 1.31 (95% CI: 0.0.90–1.90), and 1.51 (95% CI: 1.04–2.19), respectively. Hydrocarbons aHRs for Q2, Q3, and Q4 were 0.87 (95% CI: 0.64–1.18), 1.16 (95% CI: 0.90–1.49), and 1.40 (95% CI: 1.06–1.85), respectively. Increased exposure to NO, NO_2_, and hydrocarbons is associated with increased SP risk in Taiwanese children.

## 1. Introduction

Long-term ambient air pollution is associated with an increased risk of respiratory, cardiovascular and cerebrovascular diseases [1,2]. Respiratory diseases such as chronic obstructive pulmonary disease (COPD) and asthma may be exacerbated by air pollution; air pollution may also increase morbidity and mortality associated with respiratory diseases [3]. The health effects of air pollution depend on the components and sources of pollutants, which vary with countries, seasons, and times.

Spontaneous pneumothorax (SP) is defined as the spontaneous appearance of air in the pleural space of a patient. SP is caused by the rupture of blebs or emphysematous bullae that develop just beneath the pulmonary pleura. Although the pathogenic mechanisms of SP remain unclear, there is epidemiological evidence of a significant association between the risk of SP and exposure to environmental factors, such as cigarette smoke and meteorological conditions [4,5,6,7,8]. Some studies have suggested that atmospheric pressure, temperature changes, and specific weather phases may be precipitating factors in the development of SP [9,10]. Han et al. found that air pollution exposure, especially pollution containing particulate matter (PM), increased hospital visits due to SP [11]; however, a recent study by Marx et al. found no connection between exposure to NO_2_ or PM with diameters ≤10 µm (PM_10_) and primary SP but did identify an association between SP and O_3_ exposure [12]. In summary, the association between air pollution and SP remains unclear and debatable. Therefore, we conducted this nationwide, retrospective study to evaluate the effect of air pollution exposure on the risk of SP in Taiwanese children.

## 2. Patients and Methods

We conducted a population-based cohort study using the file on children (aged < 18 years) that is part of the database of citizens enrolled in the Taiwan National Health Insurance (NHI) program [13] and the Taiwan Air Quality-Monitoring Database (TAQMD), which is released by the Taiwan Environmental Protection Agency. Details on the file on children and the TAQMD have been provided in previous studies [14,15,16]. We combined the pediatric file and the TAQMD by linking the residential areas of insured with nearby 74 air-quality-monitoring stations. The clinics and hospitals in various residential areas were identified, and the study was based on the records of insured children treated for acute upper respiratory tract infections (International Classification of Diseases, Ninth Revision, Clinical Modification, ICD-9-CM code 460). This study was approved by the Research Ethics Committee of China Medical University and Hospital in Taiwan (CRREC-103-048).

This study selected a cohort of children younger than 18 years on January 1, 2000 (the index date). We excluded children with a history of pneumothorax (ICD-9-CM codes 512.0, 512.1, 512.2, 512.8) before the index date. The person-years in the follow-up period were counted for each child until they withdrew from the NHI program, expired, developed pneumothorax, or until 31 December 2012. A daily average air pollutant concentration was calculated from 2000 until the end of the observation year for each study subject. Air pollutant concentrations were grouped into four quartiles: nitric oxide (NO) concentration (Q1: <5.18 parts per billion (ppb), Q2: 5.18–8.44 ppb, Q3: 8.44–11.6 ppb, and Q4: >11.6 ppb), nitrogen dioxide (NO_2_) concentration (Q1: <18.2 ppb, Q2: 18.2–23.7 ppb, Q3: 23.7–26.9 ppb, and Q4: >26.9 ppb), and total hydrocarbons concentration (Q1: <2.29 parts per million (ppm), Q2: 2.29–2.37 ppm, Q3: 2.37–2.60 ppm, and Q4: >2.60 ppm). Confounding factors in the study were sex, age, monthly income, and urbanization level.

Age data are presented as mean ± standard deviation and were compared with different air pollutant concentration levels using one-way analysis of variance. Numbers and percentages are presented by air pollutant concentration level for categorical variables such as sex, monthly income, urbanization level, and outcome (pneumothorax); differences were assessed by the Chi-square test. The NHI has stratified all city districts and townships in Taiwan into 7 urbanization levels, based on population density (people/km^2^), proportion of residents with higher education, elderly and agricultural population, and the number of physicians per 100,000 people in each area [17]. Level 1 represented areas with a higher population density and socioeconomic status, and level 7 represented the lowest. As few people lived in the more rural areas of levels 4–7, our study grouped these areas into the level 4 group. The incidence density rate of pneumothorax (per 10,000 person-years) was calculated by air pollutant concentration level.

Univariable and multivariable Cox proportional hazard regression models were used to estimate the hazard ratios (HRs) and 95% confidence intervals (CIs) for pneumothorax at Q2–Q4 air pollutant concentrations compared with the reference point (Q1). The multivariable model was adjusted for age, sex, monthly income, and urbanization level. This type of statistical model can be used to assess the relationship between multiple variables, allowing for the assessment of independent relationships while also adjusting for potential confounders. We have used age as a continuous variable in the models, whereas sex, monthly income, and urbanization were applied as categorical variables.

All analyses were conducted using SAS software Version 9.4 (SAS Institute Inc., Cary, NC, USA), and the significance level was set at a 2-tailed *p* < 0.05.

## 3. Results

Table 1 presents data for children according to the level of exposure to NO. The daily average NO concentration was 12.7 ± 10.8 ppb (data not shown). The mean age of children located in the highest concentration areas (Q4) of NO was highest at 7.06 ± 3.66 years; 52.1% of children were exposed to Q2 levels of NO. Children exposed to the highest NO concentrations were more likely to have higher monthly incomes, live in higher urbanization areas, and have higher frequencies of pneumothorax than other groups.

The average daily NO_2_ concentration was 24.4 ± 5.57 ppb (data not shown). The mean age was highest (6.70 ± 3.55 years) among children exposed to Q4 levels of NO_2_ (Table 2). Boys accounted for 52.3% of children exposed to Q1 NO_2_ concentrations. Children exposed to Q1 levels of NO_2_ had the highest percentage of children in the lowest income group. Children exposed to Q4 NO_2_ concentrations had the highest percentage of children living in highly urbanized areas, as well as the highest frequency of pneumothorax.

The average daily total hydrocarbons concentration was 2.42 ± 0.23 ppm (data not shown). The mean age was highest (7.74 ± 3.84 years) among children exposed to Q4 concentrations of hydrocarbons, as was the percentage of girls (49.5%) (Table 3). Among children exposed to Q4 hydrocarbon levels, 49.9% lived in the most highly urbanized areas, as opposed to 6.43% in the least urbanized areas. Children exposed to Q1 levels of hydrocarbons reported a lower monthly income. Children exposed to the Q4 levels of total hydrocarbons had the highest frequency of pneumothorax among all groups.

Table 4 presents the risks of pneumothorax by the level of air pollutant concentration. The incidence rate of pneumothorax increased with increasing NO and NO_2_ concentrations. The NO adjusted hazard ratios (aHRs) for Q2, Q3, and Q4, when compared with Q1, were 1.11 (95% confidence interval CI: 0.77–1.61), 1.24 (95% CI: 0.88–1.76), and 1.66 (95% CI: 1.17–2.34), respectively. The NO_2_ aHRs for Q2, Q3, and Q4 were 1.12 (CI: 0.77–1.64), 1.31 (95% CI: 0.0.90–1.90), and 1.51 (95% CI: 1.04–2.19), respectively. Hydrocarbons aHRs for Q2, Q3, and Q4 were 0.87 (95% CI: 0.64–1.18), 1.16 (95% CI: 0.90–1.49), and 1.40 (95% CI: 1.06–1.85), respectively.

Participants exposed to Q4 levels of NO had a 1.66-fold higher risk of pneumothorax than those exposed to Q1 levels, while those exposed to Q4 levels of NO_2_ were 1.51 times more likely than those exposed to Q1 levels to suffer a pneumothorax. Those exposed to Q4 hydrocarbon levels had a 1.40-fold higher risk of pneumothorax than those exposed to Q1 levels.

## 4. Discussion

In this nationwide retrospective cohort study, the participants exposed to increasing levels of nitric oxides (NOx) and total hydrocarbons were at increased risk of spontaneous pneumothorax.

Pneumothorax is due to the spontaneous rupture of subpleural blebs or bullae [18]. Subpleural blebs and bullae are found in the lung apices at thoracoscopy and on computed tomography scanning in up to 90% of cases of SP [19], compared with 20% of controls matched for age and smoking status [20]. Even among nonsmokers with a history of pneumothorax, 81% have bullae [21]. The development of blebs or bullae may be linked to a variety of factors, including distal airway inflammation [22], hereditary predisposition [23], anatomical abnormalities of the bronchial tree [24], apical ischemia at the apices [25,26], low body mass index and caloric restriction [27], and abnormal connective tissue [28]. Air pollution contributes to worsening chronic inflammation of the lung and may eventually lead to SP. Rapid changes in environmental conditions may be a precipitating factor in the development of SP. Previous studies have suggested an association between SP and changes in meteorological conditions. One study reported increases in SP were associated with days having significantly higher wind speeds and lower atmospheric pressures [29]. In Japan, the temperature was linked to SP events [30,31]. Environmental factors such as NO_2_, ozone (O_3_), and carbon dioxide concentrations are reported to influence the onset of SP, as are viral epidemics occurring in autumn or spring (adenovirus, rhinovirus, etc.) that may increase the frequency of SP by inducing hyperreactivity cough [10,32]. Bertolaccini et al. found that SP (first episode or relapse, without age selection) occurs in clusters and is significantly more likely on warm, windy days with low atmospheric pressure and high mean NO_2_ concentrations [33,34]. However, those studies had potential limitations, such as a small study size and lack of age selection.

The term NOx describes a mixture of nitric oxide (NO) and NO_2_. Most NOx is present as NO, but this species is readily oxidized to NO_2_ by reaction with O_3_, so NOx levels are similar to standard values for NO_2_. Nitric oxides in the air can irritate the eyes, nose, throat, and lungs. It inflames the lining of the lungs, and it can reduce immunity to lung infections. This may worsen cough and wheezing, reduce lung function, and increase asthma attacks [33]. Moreover, it may also result in fluid build-up in the lungs 1 or 2 days after exposure. The health effects of hydrocarbons have been noted in occupational exposures to tetra methyl lead, benzene, and other substances. Inhalation of hydrocarbons can also cause irritation, and hydrocarbons are major contributors to eye and respiratory irritation caused by photochemical smog. A study of rats exposed to hydrocarbons revealed a history of lung edema and hemorrhagic necrosis of lung alveoli and parenchyma [34]. The evidence suggested that this was the result of damage to the antioxidant defense system with consequential loss of cell and tissue surfactant. Further animal evidence of lung pathology associated with exposure to hydrocarbons revealed the rupture of alveoli and terminal bronchioles [35]. It is not clear whether this pathology is related to hydrocarbon toxicity, the consequence of increased intraluminal pressure, or both. Both NOx and hydrocarbons in the air can damage the lung and cause bullae to rupture more easily, thereby increasing the risk of SP.

Several previous studies have indicated that exposure to air pollutants such as NO_2_, PM and O_3_ might induce systemic inflammation [11,12]. Air pollutants such as NO and hydrocarbon may enter the pleural space through various mechanisms: direct alveolar rupture (as in emphysema or necrotic pneumonia) via the lung interstitium or backward via the bronchovascular bundle and mediastinal pleura (pneumomediastinum).

In our study, we assessed participants who were younger than 18 years. To the best of our knowledge, our study is the first study in the literature to evaluate the link between childhood SP and air pollution. As COPD does not occur in individuals in this age group, and these participants had no history of asthma, confounding factors associated with these two diseases were excluded. The confounding factors considered in this study were age, sex, monthly income, and urbanization level. We used a multivariable model to assess the relationships between multiple variables, which allowed us to assess independent relationships while adjusting for these four confounders. Our findings showed that these confounding factors rarely influenced the outcomes of our study, with male adolescence (age of 10–18 years) identified as the only factors likely to increase the risk of subsequent SP events, which has been previously identified.

We defined the areas of the subjects according to the location of the clinics where they most frequently sought treatment for acute upper respiratory tract infections. Furthermore, we used urbanization as a covariate in a multivariate analysis model, in addition to controlling for the influences of available medical resources and social status. Although differences in urbanization levels among towns throughout Taiwan were considered, potential bias may have resulted from defining the active area according to the location of medical institutions where residents sought acute upper respiratory tract infection treatment. Heathy residents are more likely to be exposed to the lowest levels of air pollution, and this may have led to the underestimation of SP risk.

The main limitation of this study was the lack of detail on the clinical histories, including body weight, height, and smoking history. It is known that smoking is a risk factor for SP, but we were unable to determine if these patients had a smoking history or if they had been exposed to smoking environments. The severity of SP was also unknown in our study, or if it was related to the severity of air pollution. Meteorological conditions such as temperature, atmospheric pressure, and rainfall may be related to the onset of SP but were not recorded in the LHID. Therefore, further investigation is warranted.

## 5. Conclusions

In summary, we observed an increasing trend in the relationship between air pollution levels and the risk of SP among children. Exposure to the highest level of NOx and hydrocarbons may increase the risk of SP in the Taiwanese pediatric population. Comparisons of the impacts of air pollution between children and adults represent a potential direction for future study.

## Figures and Tables

**Table 1:** Characteristics of participants exposed to various annual average concentrations of nitric oxide.

	Nitric Oxide (n = 255,380)								
	Quartile 1 (Q1) (Lowest) n = 34,316		Quartile 2 (Q2) n = 56,629		Quartile 3 (Q3) n = 85,060		Quartile 4 (Q4) (Highest) n = 79,375		*p*-Value
Variable	n	%	n	%	n	%	n	%	
Age (mean, SD)	6.24	3.38	6.03	3.05	6.19	3.25	7.06	3.66	<0.001
Boys	17,773	51.8	29,497	52.1	44,085	51.8	40,487	51.0	<0.001
Monthly income (NTD)									<0.001
<14,400	29,415	85.7	48,268	85.2	72,151	84.8	64,004	80.6	
14,400–18,300	3308	9.64	5992	10.6	9669	11.4	10,586	13.3	
18,300–21,000	722	2.10	1161	2.05	1553	1.83	2151	2.71	
≥21,000	871	2.54	1208	2.13	1687	1.98	2634	3.32	
Urbanization level									<0.001
1 (highest)	4528	13.2	11,994	21.2	30,638	36.0	37,804	47.6	
2	12,224	35.6	17,210	30.4	29,413	34.6	22,852	28.8	
3	3508	10.2	14,780	26.1	16,822	19.8	13,317	16.8	
4 (lowest)	14,056	41.0	12,645	22.3	8187	9.62	5402	6.81	
Outcome									
Pneumothorax (ICD-9-CM 512.0, 512.8)	43	0.13	83	0.15	140	0.16	173	0.22	<0.001

ICD-9-CM: International Classification of Diseases, Ninth Revision, Clinical Modification; NTD: new Taiwan dollar; SD: standard deviation.

**Table 2:** Characteristics of participants exposed to various annual average concentrations of nitrogen dioxide.

	Nitric Dioxide (n = 255,380)								
	Quartile 1 (Q1) (Lowest) n = 29,082		Quartile 2 (Q2) n = 70,475		Quartile 3 (Q3) n = 72,168		Quartile 4 (Q4) (Highest) n = 83,655		*p*-Value
Variable	n	%	n	%	n	%	n	%	
Age (mean, SD)	6.05	3.12	6.24	3.33	6.46	3.31	6.70	3.55	<0.001
Boys	15,221	52.3	36,631	52.0	37,315	51.7	42,675	51.0	<0.001
Monthly income (NTD)									<0.001
<14,400	24,975	85.9	59,736	84.8	60,077	83.3	69,050	82.5	
14,400–18,300	2909	10.0	7594	10.8	8986	12.5	10,066	12.0	
18,300–21,000	587	2.02	1435	2.04	1533	2.12	2032	2.43	
≥21,000	611	2.10	1710	2.43	1572	2.18	2507	3.00	
Urbanization level									<0.001
1 (highest)	3740	12.9	14,772	21.0	23,335	32.3	43,117	51.5	
2	7652	26.3	25,083	35.6	26,300	36.4	22,664	27.1	
3	4730	16.3	14,864	21.1	16,454	22.8	12,379	14.8	
4 (lowest)	12,960	44.6	15,756	22.4	6079	8.42	5495	6.57	
Outcome									
Pneumothorax (ICD-9-CM 512.0, 512.8)	39	0.13	103	0.15	127	0.18	170	0.20	<0.001

ICD-9-CM: International Classification of Diseases, Ninth Revision, Clinical Modification; NTD: new Taiwan dollar; SD: standard deviation.

**Table 3:** Characteristics of participants exposed to various annual average concentrations of total hydrocarbons.

	Total Hydrocarbons (n = 255,380)								
	Quartile 1 (Q1) (Lowest) n = 67,078		Quartile 2 (Q2) n = 50,365		Quartile 3 (Q3) n = 85,457		Quartile 4 (Q4) (Highest) n = 52,480		*p*-Value
Variable	n	%	n	%	n	%	n	%	
Age (mean, SD)	5.51	2.60	5.54	2.73	6.88	3.62	7.74	3.84	<0.001
Boys	35,017	52.2	26,114	51.9	44,211	51.7	26,500	50.5	<0.001
Monthly income (NTD)									<0.001
<14,400	59,734	89.1	44,650	88.7	69,566	81.4	39,888	76.0	
14,400–18,300	5685	8.48	4133	8.21	11,170	13.1	8567	16.3	
18,300–21,000	885	1.32	769	1.53	2126	2.49	1807	3.44	
≥21,000	774	1.15	813	1.61	2595	3.04	2218	4.23	
Urbanization level									<0.001
1 (highest)	18,901	28.2	11,346	22.5	28,534	33.4	21,683	49.9	
2	16,353	24.4	19,771	39.3	29,906	35.0	15,669	29.9	
3	15,490	23.1	8003	15.9	17,680	20.7	7254	13.8	
4 (lowest)	16,334	24.4	11,245	22.3	9337	10.9	3374	6.43	
Outcome									
Pneumothorax (ICD-9-CM 512.0, 512.8)	106	0.16	68	0.14	153	0.18	112	0.21	<0.01

ICD-9-CM: International Classification of Diseases, Ninth Revision, Clinical Modification; NTD: new Taiwan dollar; SD: standard deviation.

**Table 4:** Comparisons of pneumothorax incidence and associated hazard ratios by average annual concentration of air pollutants.

	Pollutant Levels	Event	PY	IR	cHR	95% CI	aHR	95% CI
NO								
Q1: <5.18 ppb	34,316	43	375,702	1.14	Ref.		Ref.	
Q2: 5.18–8.44 ppb	56,629	83	634,501	1.31	1.14	(0.79, 1.64)	1.11	(0.77, 1.61)
Q3: 8.44–11.6 ppb	85,060	140	939,106	1.49	1.30	(0.93, 1.83)	1.24	(0.88, 1.76)
Q4: >11.6 ppb	79,375	173	816,112	2.12	1.99	(1.42, 2.78) ***	1.66	(1.17, 2.34) **
NO_2_								
Q1: <18.2 ppb	29,082	39	324,172	1.20	Ref.		Ref.	
Q2: 18.2–23.7 ppb	70,475	103	773,695	1.33	1.11	(0.77, 1.60)	1.12	(0.77, 1.64)
Q3: 23.7–26.9 ppb	72,168	127	781,264	1.63	1.39	(0.97, 1.99)	1.31	(0.90, 1.90)
Q4: >26.9 ppb	83,655	170	886,289	1.92	1.66	(1.17, 2.35) **	1.51	(1.04, 2.19) *
Total Hydrocarbons								
Q1: <2.29 ppm	67,078	106	782,116	1.36	Ref.		Ref.	
Q2: 2.29–2.37 ppm	50,365	68	584,105	1.16	0.86	(0.63, 1.17)	0.87	(0.64, 1.18)
Q3: 2.37–2.60 ppm	85,457	153	890,724	1.72	1.40	(1.09, 1.79) **	1.16	(0.90, 1.49)
Q4: >2.60 ppm	52,480	112	508,476	2.20	1.95	(1.49, 2.54) ***	1.40	(1.06, 1.85) *

*: <0.05; **: <0.01; ***: <0.001.

## Data Availability

The data are not publicly available due to ethical restrictions.
